# Abstracts

**Published:** 1915-12

**Authors:** 


### ABSTRACTS.

---

Have you ever felt that this department of the BUFFALO MEDICAL JOURNAL was lacking in abstracts of your specialty, or that such as were published lacked the expert judgement which you yourself could have exercised in selection and preparation? If not, you are more charitable than the editor has been to himself. If so, will you assist in abstracting from a few journals, and thus make this department more representative and more valuable? Incidentally, there are few forms of study more helpful to the worker than this.

---

We have constantly in mind the fear of selecting for Abstracts articles that may interest the reviewer but not the general reader, and also of failing to preserve the happy mean between too great length and so great brevity as to omit the gist of the article. With regard to the former, we are pleased to note that the Medical Record has considered worthy of editorial treatment, two of the subjects which we have recently published in abstract form: Achalasia of the Cardia and Determination of Plasma and Blood Volumes. To prevent misunderstanding, we wish to state explicitly that the Record could not even have had attention called to these subjects from our Abstracts, owing to date of publication. The Nashville Journal of Medicine and Surgery also reprints with credit, our Abstracts upon Marion's Advocacy of Nephropexy, with comments, as well as one on the Treatment of Tuberculosis by Compression, without comments. While we appreciate the courtesy, and recognise the propriety of assigning responsibility in the former case, since editorial comment was added, we do not ask credit for any news item or abstract representing nothing but literary drudgery, even if reprinted from our original wording. For example, we abstracted Marion's article from a translation without credit to the intermediate source, so on the ground that the mutual exchange of information is the reason for exchange of journals, although in any case in which we have simply clipped from an exchange, our rule is to give due credit even for the labor saved.

